# Pharmacokinetics of mebendazole in plasma and cerebrospinal fluid following a single oral dose in healthy dogs

**DOI:** 10.3389/fvets.2023.1231769

**Published:** 2023-08-28

**Authors:** Amy B. Yanke, Kendall E. Day, Amanda R. Taylor, Crisanta Cruz-Espindola, Dawn M. Boothe

**Affiliations:** ^1^Department of Clinical Sciences, College of Veterinary Medicine, Auburn University, Auburn, AL, United States; ^2^BluePearl Pet Hospital North Dallas, Lewisville, TX, United States; ^3^Southeast Veterinary Neurology, Boynton Beach, FL, United States; ^4^Department of Anatomy, Physiology and Pharmacology, College of Veterinary Medicine, Auburn University, Auburn, AL, United States

**Keywords:** dog, glioma, mebendazole, pharmacokinetics, plasma, cerebrospinal fluid

## Abstract

Novel therapies are needed for treatment of gliomas. Mebendazole previously demonstrated anti-neoplastic effects on canine glioma cell lines at *in vitro* mean inhibitory concentrations (IC_50_) of 10 ng/mL. Our study aimed to titrate the oral dose of mebendazole necessary to achieve concentrations ≥10 ng/mL in cerebrospinal fluid (CSF) of healthy dogs. We hypothesized that an oral dose up to 200 mg/kg would be necessary. Phase one was a dose titration study using a total of 6 mixed breed dogs that described dose vs. plasma concentrations for 72 h after single oral dosing of either 50 mg/kg (*n* = 2), 100 mg/kg (*n* = 2), or 200 mg/kg (*n* = 2). Based on phase one, phase two dogs (total of 9) received 100 mg/kg (*n* = 4) or 200 mg/kg (*n* = 5) orally and blood samples were collected intermittently for 60 h with CSF samples collected intermittently for 24 h. Mebendazole was quantitated in plasma and CSF using high performance liquid chromatography. Median peak plasma concentrations (Cmax) were reached at 7 ± 2 h (100 mg/kg) of 220 ng/mL (81, 283) and at 15 ± 4 h (200 mg/kg) of 147 ng/ml (112, 298). The respective area under the curve (AUC: ng/ml/h) reported as a median was 2,119 (1,876, 3,288) vs. 3,115 (1,559, 4,972). Median plasma concentrations (ng/ml) for 100 vs. 200 mg/kg were 47 (32, 52) vs. 65 (35, 104), respectively. For CSF, the median value for Cmax (at 100 mg/kg vs. 200 mg/kg) was 8 (2, 28) vs. 21 (12, 27) and AUC was 87 (22, 157) vs. 345 (92, 372), respectively. Relative bioavailability in CSF vs. plasma was 4 to 10%. Although several animals demonstrated clinical signs indicative of gastrointestinal upset [i.e., vomiting (*n* = 2), diarrhea (*n* = 2), or both (*n* = 1)], these events were not considered serious. The *in vitro* IC_50_ for gliomas can be reached in CSF at 100 mg/kg (*n* = 1), however a 200 mg/kg dose yielded more consistent concentrations.

## 1. Introduction

Gliomas are primary brain tumors arising from glial cells that differentiate into astrocytomas, oligodendrogliomas, or ependymomas. These tumors are common in both humans and canines (1–4). Canine gliomas are typically presumptively diagnosed based on magnetic resonance imaging (MRI) features of the lesion, as definitive diagnosis with a biopsy is not commonly obtained (5). As such, their aggressive nature and intra-axial location limit the effectiveness of available treatment options in both veterinary and human medicine which often involves multiple treatment modalities including surgery, radiation therapy, and/or chemotherapeutic agents such as temozolomide (5–8). Despite the limited effectiveness of these treatment options, owners of dogs with presumed glial tumors are becoming more interested in treatment options for their canine companions, particularly those that are less invasive and cost conscious to allow for increased survival and preservation of quality of life. Due to this interest, continued research into novel therapies for gliomas is an important focus that has a potential to benefit both human and veterinary medicine.

Benzimidazoles (BZDs), such as fenbendazole and mebendazole, were originally developed as anthelmintics for use in human and veterinary medicine (9–13). Recently it has been discovered that BZDs have multiple anti-neoplastic properties against various cancer cell lines *in vitro* as well as *in vivo* (13–21). Their primary mode of action targets tubulin, one of the main building blocks of a cell that is crucial for cell division (13, 14). More specifically, BZDs inhibit the polymerization of tubulin and disrupt the formation of microtubules which leads to cell arrest (14–16). Additional anti-neoplastic properties of BZDs include inhibition of the hedgehog pathway, inhibition of kinases (such as MAPK14/p38a), and antiangiogenic effects (15–23). In recent human literature, BZDs have been found to inhibit the growth and development of several cancers including non-small-cell lung cancers, adrenocortical carcinoma, chemoresistant melanoma, colon cancer, hepatocellular carcinoma, and central nervous system (CNS) neoplasms such as glioblastoma, other gliomas, and medulloblastoma (13–26).

Previous research has demonstrated *in vitro* anti-tubulin and anti-neoplastic effects of fenbendazole and mebendazole on three canine glioma cell lines, establishing a mean inhibitory concentration (IC_50_; MIC) for each drug (12). The *in vitro* IC_50_ of mebendazole was found to be 10 ng/ml vs. approximately 150 ng/ml for fenbendazole (12), demonstrating mebendazole to be a more potent drug compared to fenbendazole. Additionally, previous studies have shown that dosages of 11–110 times the anthelmintic dose of 22 mg/kg/day of mebendazole given to dogs daily for 2 months produced no adverse reactions or effects on liver function (10). Unpublished data from a previous study using fenbendazole in our lab revealed profound gastrointestinal upset in the cohort of healthy dogs. These findings make mebendazole a better drug of choice for additional study as an anti-neoplastic agent.

Documenting mebendazole concentrations in the cerebrospinal fluid (CSF) of healthy dogs and comparing those to what has been shown to produce increased glioma cell line death *in vitro* is a crucial step in the formulation of a clinical trial in canine glioma patients. Without showing that mebendazole is capable of crossing the blood brain barrier and achieving detectable concentrations in healthy canine CSF, there is little support that BZDs would have any use as a potential therapy for canine gliomas. The objectives of this study were to describe the time course of mebendazole concentrations in the plasma and CSF when administered orally to healthy dogs, and subsequently, to determine an oral dose of mebendazole necessary to achieve and maintain concentrations predicted to be therapeutic for the treatment of gliomas in dogs. We hypothesized that 1) canine plasma mebendazole concentrations will increase in a dose dependent manner and that 2) an oral dose up to 200 mg/kg will achieve *in vivo* concentrations of at least 10–20 ng/ml in the CSF of a healthy dog, which was considered therapeutic concentrations for the treatment of canine gliomas based on the previous *in vitro* study (12).

## 2. Materials and methods

### 2.1. Animals

A total of six (age range: 2–10 years) healthy female (4 intact, 2 spayed) mixed breed dogs were acquired from a laboratory purpose-bred breeding colony at the Auburn University College of Veterinary Medicine (AUCVM) for use in phase 1 of this study. Three additional healthy male intact mixed breed dogs (age range: 2–8 years) were acquired in addition to the original six female dogs (*n* = 9) for use in phase 2 of this study. Upon entry into the hospital on Day 0 of each phase of the study, dogs were given ≥12 h to acclimate prior to starting blood collection (and CSF collection – phase 2 only). All dogs were apparently healthy and had unremarkable physical and neurological examinations (performed by the same investigators throughout the entirety of the study) prior to drug administration. A complete blood count (CBC), serum chemistry panel, and urinalysis were additionally performed prior to drug administration to assess baseline health status. Exams were performed daily until the end of blood collection for each phase of the study. Monitoring of vitals (temperature, heart rate, respiratory rate), mentation, and any signs of pain or discomfort (e.g., vocalization, elevation in vitals, etc.) were assessed every 6–8 h during both phases of the study. The dogs were hospitalized during the collection phases of the study and were returned to the breeding colony kennels between phases of the study and ultimately at the conclusion of phase 2 of the study. All procedures were approved by the Auburn University Institutional Animal Care and Use Committee.

### 2.2. Vascular access

On Day 0 of each phase of the study, all dogs had intravenous catheters placed in either the left or right cephalic veins. All dogs were then heavily sedated with dexmedetomidine (5 μg/kg) and butorphanol (0.2 mg/kg) for placement of jugular catheters, as described elsewhere (27), to provide vascular access for collection of blood samples. Jugular catheters were placed 12–24 h prior to the initiation of sample collection. Catheters were flushed with heparinized sterile saline (0.9% NaCl) once daily and subsequently flushed with sterile saline and monitored for patency and cleanliness every 6 h. Routine bandaging of the catheter was implemented and replaced as indicated by sample collection design or as needed based on the patient. Catheters were removed immediately after the last blood sample was obtained and catheter sites were inspected for any evidence of infection. Pressure bandages were placed over the catheter site for 30 min following removal and were subsequently removed prior to return to the colony kennels.

### 2.3. Mebendazole administration

Mebendazole was supplied by a collaborator, Dr. Gregory Riggins, a Professor of Neurosurgery and Oncology at Johns Hopkins University, in a powdered formulation. While it still retains FDA approval for use in dogs, mebendazole is no longer readily commercially available in the United States of America at the present time. Based on known therapeutic dosing (10, 11) and the study’s aimed CSF concentration, a dose of 50 mg/kg, 100 mg/kg, or 200 mg/kg was compounded into capsules for each dog (one dose per dog as randomly assigned for each phase of the study) by the Auburn University Small Animal Veterinary Teaching Hospital Pharmacy. During phase 1 (Dose Determination, *n* = 6 total), two dogs were randomly assigned to receive either a 50, 100, or 200 mg/kg single oral mebendazole dose. During phase 2 (Pharmacokinetics, *n* = 9 total), dogs were randomized to receive a single oral mebendazole dose of either 100 mg/kg (*n* = 4) or 200 mg/kg (*n* = 5). At the time of administration in both studies, a minimal amount of food was used to allow for ingestion of the dose; animals were not given a full meal until 6 h post-administration during phase 1 and not until roughly 12–14 h post-administration due to the frequent anesthetic periods during phase 2 to reduce the risk of regurgitation/aspiration.

### 2.4. Blood collection

During phase 1, blood samples were collected from all dogs at times 0, 0.5, 1, 2, 4, 6, 8, 12, 16, 20, 24, 48, and 72 h post mebendazole administration. During phase 2, this was similar, but concluded at 60 h (instead of 72 h) post-administration. The described “three-syringe technique” was used to sterilely collect blood at each collection time and the catheters were flushed with 3 ml of sterile saline (0.9% NaCl) afterwards (28). Blood samples did not exceed 6 ml at each collection time. Packed cell volume and total solids were monitored every 24 h to ensure we did not exceed 7% blood volume sampled of body weight over 24 h. Blood samples were initially stored in Sarstedt S-Monovette® Neutral Z/4.9 ml tubes. Blood samples were stored at 0°C for no more than 10–20 min prior to centrifugation. Plasma was properly obtained via centrifugation for 5 min at 1,500 × *g*. Plasma samples were transferred via plastic pipette to a Sarstedt screw cap 1.5 ml micro tube and were then placed into −80°C for storage until processing was performed. Samples were stored for approximately 3 months.

### 2.5. CSF collection

During phase 2, CSF samples were collected from all dogs at times 1, 2, 3, 6, 12, and 24 h post mebendazole administration. All dogs were placed under general anesthesia for CSF collection. Dogs received an injection of butorphanol (0.2 mg/kg) and dexmedetomidine (5 μg/kg) as a pre-medication. Propofol (3–6 mg/kg IV to effect) was used for induction and the dogs were then intubated using an endotracheal tube. Dogs were maintained on isoflurane in oxygen titrated to effect. Dogs were kept under a single anesthetic event for collection times 1–3 h. For samples at hour 6, 12, and 24, they were placed under separate anesthetic events for a total of 4 anesthetic events over a time period of 24 h. A sterile 22-gauge, 1.5-inch spinal needle was used to obtain approximately 1 ml of CSF from the atlantooccipital junction at each collection time. This volume of CSF removed was deemed safe as canine CSF production has been determined to be 0.047 ml/min or around 68 ml/day (29). When there was evidence of gross blood contamination, a few drops of CSF were discarded until the fluid became clear and then collection began. CSF samples were not analyzed for protein, red blood cell, or white blood cell counts as part of this study. Samples were collected into a Sarstedt screw cap 1.5 ml micro tube and stored at 0°C for no more than 10–20 min prior to being placed into −80°C for storage until processing was performed. Samples were stored for approximately 3 months.

### 2.6. Pharmacokinetic analysis

Dog plasma and cerebrospinal fluid (CSF) were analyzed for mebendazole concentrations by high performance liquid chromatography (HPLC) with ultraviolet (UV) detection (30–33). The HPLC system consisted of a Waters 2,695 separation module and a 2,489 UV–Visible detector (Waters Corporation™, Milford, MA, USA). Separation was achieved with a Gemini C6, 5 μm, 150 × 3 mm column (Phenomenex®, Torrance, CA, USA) at 40°C (31–35). The mobile phase consisted of 75:25, 20 mM Ammonium formate buffer (pH adjusted to 3.0 w/formic acid):Acetonitrile (VWR®, Radnor, PA, USA) with the flow rate set to 1.5 ml/min (30, 34). The retention time for mebendazole was 6.8 min and UV absorbance was monitored at 314 nm (30–33). The standard curve was generated ranging from 10 to 1,000 ng/ml for canine plasma, and 5 to 500 ng/ml for CSF by fortifying canine plasma and saline, respectively, with known amounts of mebendazole (Sigma-Aldrich®, St. Louis, MO, USA) reference standard and accepted if the coefficient of determination (r^2^) was at least 0.99 and the predicted concentrations were within ±10% of the actual concentrations (30). Briefly, mebendazole was quantitated in canine plasma and CSF based on modifications of previously developed assays (30–35). For plasma and CSF samples, 1,000 and 700 μl of acetonitrile was added to tubes containing 500 and 350 μl of canine plasma and CSF, respectively (30, 31). The contents of each tube were mixed vigorously for 30 s through vortexing, then subjected to centrifugation for 10 min at 16,000 × *g*. The clear supernatant was transferred to a clean glass tube, then evaporated to dryness under a gentle stream of nitrogen for 40 min at 45°C (31). The residue was reconstituted with 250 μl of mobile phase, vortexed for 20 s, and then the solution was centrifuged at 16,000 × *g* for 5 min. The supernatant was transferred into the vial, and 100 μl were injected into the chromatographic system (30–32, 35).

The linear correlation coefficient for mebendazole in canine plasma and CSF was 0.999. The limit of detection was 5 and 2.5 ng/ml for canine plasma and cerebrospinal fluid (CSF), respectively. The lower limit of quantification was 10 and 5 ng/mL for canine plasma and CSF, respectively. The precision (CV %) for mebendazole in canine plasma at 14, 26, 60, 120, and 800 ng/mL was 4.37, 2.69, 1.16, 5.90, and 1.51%, respectively. The accuracy (% recovery) for mebendazole in canine plasma at 14, 26, 60, 125, and 800 ng/mL was 104.34, 99.40, 99.38, 100.28, and 101.64%, respectively. The precision (CV %) for mebendazole in canine CSF at 5, 15, 75, and 300 ng/mL was 7.70, 2.63, 2.44, and 2.11%, respectively. The accuracy (% recovery) for mebendazole in canine CSF at 5, 15, 75, and 300 ng/mL was 106.55, 100.10, 99.83, and 99.25%, respectively.

Plasma and CSF mebendazole concentration vs. time data were subjected to non-compartmental analysis using computer software (Phoenix® WinNonLin® V7, Pharsight, Cetara, Princeton, NJ, USA). Area under the curve (AUC) to infinity was determined using the log-linear trapezoidal method. The actual maximum concentration (Cmax) occurring at time to maximum concentration (Tmax) was recorded. The slope of the terminal component of the drug vs. time curve was based on non-linear regression. Because mebendazole was not given intravenously, the terminal component could not be confirmed to total elimination and thus both the elimination rate constant and half-life were reported as disappearance. Half-life was reported as harmonic mean + pseudostandard deviation. Furthermore, neither clearance (CL) nor volume of distribution (Vd) could be determined. Other parameters included mean residence time (MRT) and the percentage of the AUC that was extrapolated from the terminal component of the curve. The relative bioavailability (percentage) of mebendazole in CSF to plasma was calculated based on the ratio of the AUC (AEV1/AEV2).

### 2.7. Statistical analysis

The Kolmogorov–Smirnov test was used to determine normality between data sets. Descriptive statistics were reported as mean ± standard deviation (SD) or median and range (minimum, maximum) when not normally distributed. Comparisons were made between 100 and 200 mg/kg dosages for either plasma or CSF (and between these two sources) for key pharmacokinetic parameters using student *t*-tests and between CSF and plasma using a paired *t*-test. Differences were considered statistically significant at *p* ≤ 0.05.

## 3. Results

### 3.1. Phase 1 (dose determination)

**Figure 1:**
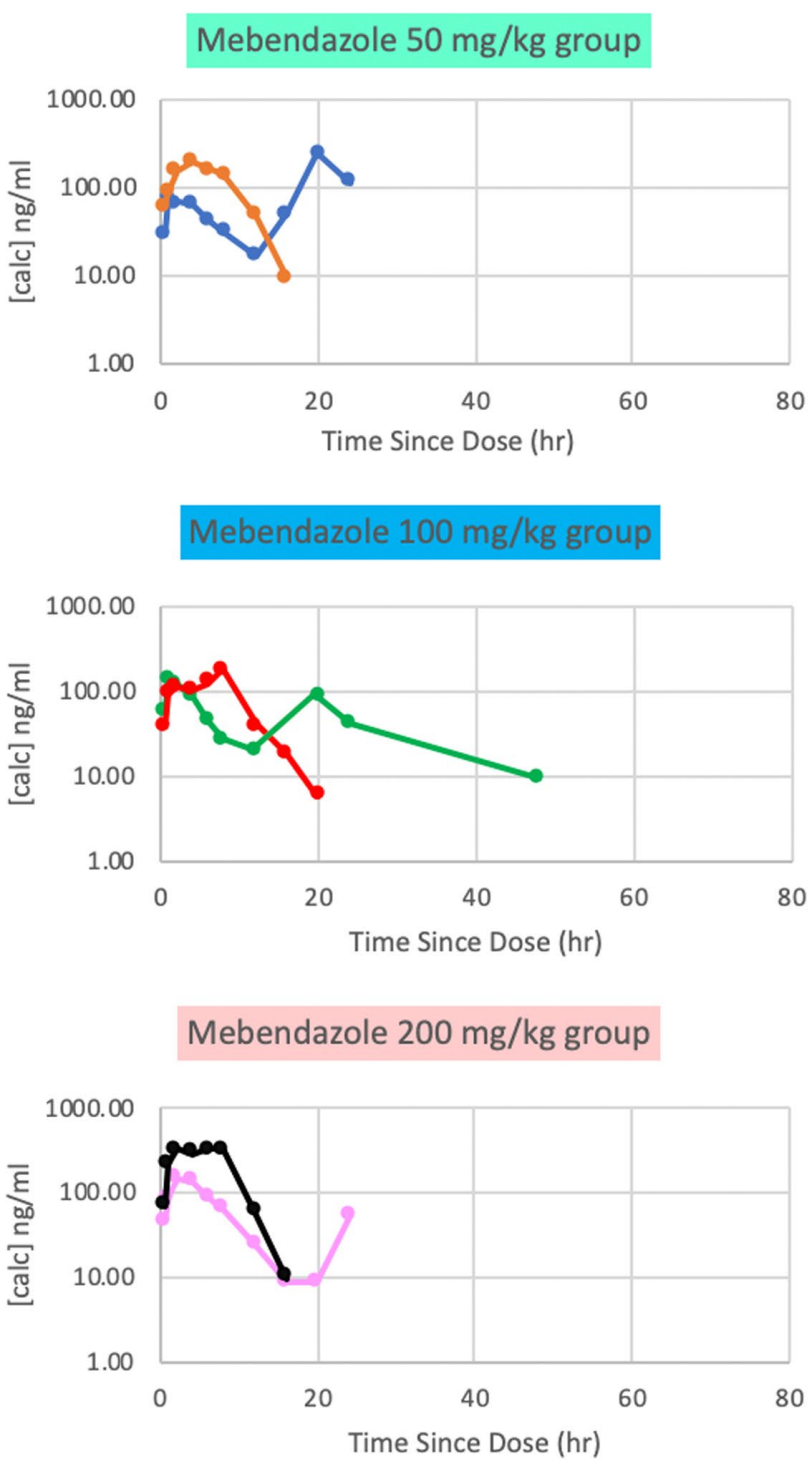
Phase 1 plasma mebendazole concentrations following a single 50 mg/kg, 100 mg/kg and 200 mg/kg oral dose, respectively. Peak plasma concentrations were at variable time points between doses.

Plasma mebendazole concentrations were detected in all dosing groups, with peak plasma concentrations (Cmax [ng/ml] (median; range) at Tmax [hrs]) reported for each respective dose being 132 ng/ml (65; 10, 235) at 3 h (50 mg/kg), 161 ng/ml (57; 6, 181) at 5 h (100 mg/kg), and 241 ng/ml (78; 9, 333) at 2 h (200 mg/kg). These findings appeared to be dose-dependent. Variability was marked among paired subjects for each dose, and concentrations dropped below the limits of detection by 24 h at all doses (Figure 1). Based on this data, phase 2 of the study was implemented at a single dose of either 100 mg/kg or 200 mg/kg to maximize plasma and CSF concentrations.

### 3.2. Phase 2 (pharmacokinetics)

**Figure 2:**
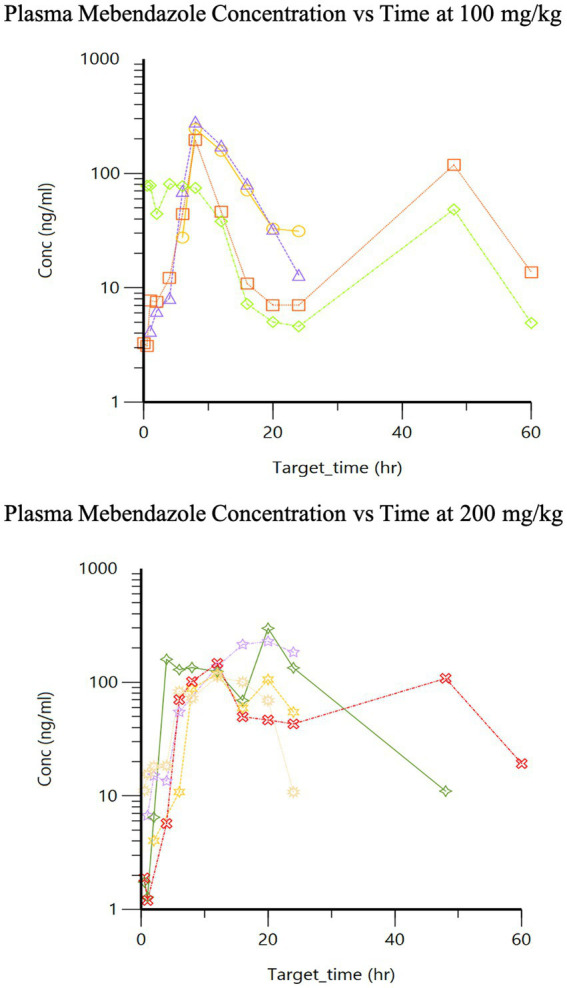
Phase 2 plasma mebendazole concentrations following a single 100 mg/kg and 200 mg/kg oral dose, respectively.

**Figure 3:**
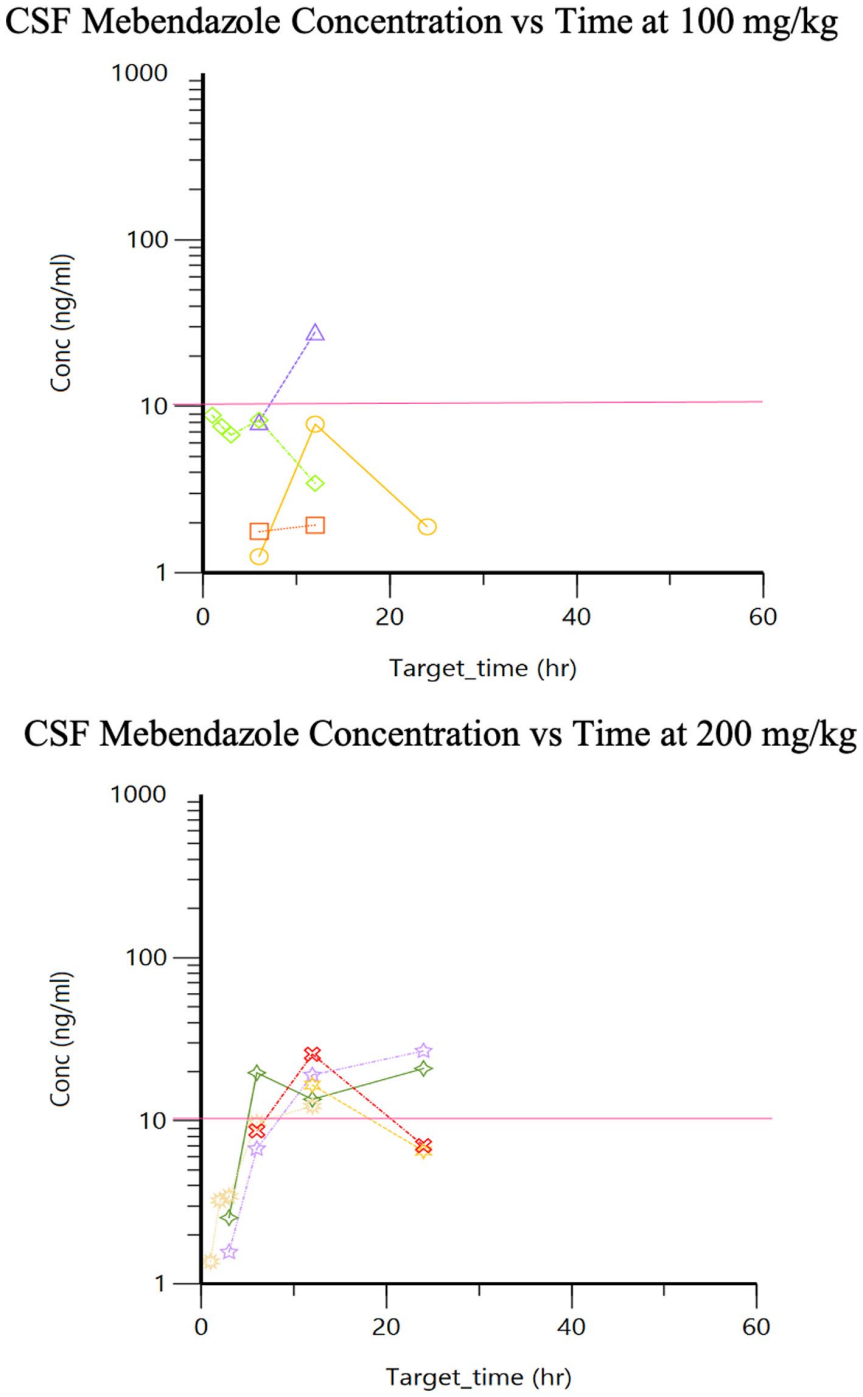
Phase 2 CSF mebendazole concentrations following a single 100 mg/kg and 200 mg/kg oral dose, respectively (pink line is target concentration, ≥10 ng/ml).

The pharmacokinetics for each dose during Phase 2 are listed in Table 1. Drug concentration vs. time graphs for 100 vs. 200 mg/kg doses achieved in plasma (Figure 2) and CSF (Figure 3) are provided. Significant differences in plasma samples between the two doses were limited to Tmax (*p* = 0.01). Plasma Tmax was 7 +/− 2 vs. 15 +/− 4 (with 100 vs. 200 mg/kg dose, respectively). For CSF, significant differences were limited to the area under the curve (AUC; *p* = 0.016). Relative bioavailability (%) of mebendazole in CSF compared to plasma was lower at 100 mg/kg (4 ± 3) compared to 200 mg/kg (10 ± 5), which was trending towards significance (*p* = 0.06). AUC revealed a difference in plasma and CSF concentrations however a moderate amount of variability was noted based on SD information comparing plasma and CSF. The Cmax_D (Cmax adjusted per dose) was higher in plasma at the 200 mg/kg vs. 100 mg/kg dose (*p* = 0.04).

**Table 1:** Pharmacokinetic results for mebendazole after oral administration of either 100 mg/kg (*n* = 4) or 200 mg/kg (*n* = 5) in apparently healthy dogs (*n* = 9) reported in plasma and cerebrospinal fluid (CSF).

Parameter	Plasma [100 mg/kg; (*n* = 4)]	Plasma [200 mg/kg; (*n* = 5)]	*p* value (100 vs. 200 plasma)	CSF [100 mg/kg; (*n* = 4)]	CSF [200 mg/kg; (*n* = 5)]	*p* value (100 vs. 200 CSF)	*p* value (all-plasma vs. CSF)	*p* value (dosed plasma vs. CSF) 100, 200
	Mean ± SDMedian (Min, Max)	Mean ± SDMedian (Min, Max)	Mean ± SDMedian (Min, Max)	Mean ± SDMedian (Min, Max)
AUC (hr*ng/ml)	2,119 (1876, 3,288)	3,115 (1,559, 4,972)	0.40	87 (22, 157)	345 (92, 372)	0.016	0.00	0.01, 0.01
Relative bioavalability (%) – CSF to Plasma	n/a	n/a	n/a	4 ± 3	10 ± 5	n/a	n/a	0.06
Cmax (ng/ml)	220 (81, 283)	147 (112, 298)	0.73	8 (2, 28)	21 (12, 27)	0.18	0.00	0.02, 0.01
Cmax_D (kg*ng/ml/mg)	2.2 (0.8, 2.8)	0.7 (0.6, 1.5)	0.04	0.08 (0.02, 0.2)	0.1 (0.06, 0.13)	0.79	0.00	0.02, 0.01
Cavg (ng/ml)	47 (32, 52)	65 (35, 104)	0.18	n/a	n/a	n/a	n/a	n/a
Clast (ng/ml)	13 (5, 31)	19 (11, 184)	0.32	9 ± 13	12 (6, 27)	0.44	0.18	0.51, 0.25
MRT (hr)	13 (12, 39)	74 (13, 93)	0.21	n/a	n/a	n/a	n/a	n/a
Tmax (hr)	7 ± 2	15 ± 4	0.01	9 ± 5	17 ± 7	0.11	0.06	0.27, 0.17

Mean ± standard deviation (SD) vs. Median (Minimum, Maximum) reported as appropriate pending normal distribution of data. *p* values were considered significant if *p* ≤ 0.05. AUC, area under the curve; Cmax, maximum (peak) concentration; Cmax_D, maximum concentration divided by dose administered; Cavg, average drug concentration; Clast, drug concentration at the last measured time point; MRT, mean residence time. Tmax, time to reach maximum concentration (Cmax).

No profound adverse events were noted for these healthy dogs receiving mebendazole during this study. During phase 1, one dog developed stool with specks of frank blood and another had one episode of mucoid diarrhea, which resolved without any further interventions. Five of the six dogs were noted to pass green stool at least once in the course of the study post-drug administration, related to the green color of the gelatin capsules used for drug administration. During phase 2, mild gastrointestinal upset was noted in five total dogs characterized by diarrhea (*n* = 2, both in 200 mg/kg group), vomiting (*n* = 2, one in 100 mg/kg group and one in 200 mg/kg group), with an additional dog from the 100 mg/kg group having both vomiting and diarrhea); all resolving without further treatment. For those that experienced vomiting, one vomited at the time of induction (~20 min) post-administration however no traces of the medication were grossly observed in the vomitus. The other episodes were several hours post-administration (7 h vs. 12 h+). The episodes of diarrhea occurred at 8-, 19- and 36-h post-administration, respectively. In phase 2, six out of nine dogs developed at least one episode of green stool during the blood collection period.

## 4. Discussion

This study demonstrates a dose concentration relationship for mebendazole after oral administration in healthy dogs and that the *in vitro* IC_50_ (0.03–0.08 μM, i.e., 10–20 ng/ml) for gliomas (12) can be reached in CSF at 100 mg/kg (*n* = 1 out of 4 in this study), although 200 mg/kg may allow for more consistent (i.e., ≥10 ng/ml) therapeutic benefit. A dose-dependent change in area under the curve (relative bioavailability) could not be demonstrated for either plasma or CSF because the dogs only received one dose of the drug (both doses must be given to determine relative bioavailability for each dog); numerically however, based on comparison of mean values, mebendazole AUC was higher in plasma and especially CSF when dogs were dosed at 200 mg/kg vs. 100 mg/kg. There was pronounced individual variability preventing the ability to generate a dose dependent curve given the small sample population.

The second peak demonstrated in our plasma concentration vs. time curves (Figure 2) after oral administration may represent individual variations of metabolism or likely a second pass metabolic process (i.e., enterohepatic circulation). Additional time points to evaluate for further elimination may be of benefit. It is also unclear how the effects of anesthesia on gastrointestinal motility or food could play a role in the total absorption and/or clearance of this drug in our study. Unpublished data from a previous study using fenbendazole in our lab may suggest better absorption with food. All dogs were withheld a full meal for the first 6 h during the sample collection of the first phase of the study and until after a minimum of 12 h for the second phase of the study trying to remove the effects of food on drug metabolism. Furthermore, a total of 5 dogs experienced gastrointestinal upset (*n* = 2 vomiting, *n* = 2 diarrhea, *n* = 1 both); however, these dogs had the highest or next to highest area under the curve for their dosing groups, suggesting this did not negatively influence the absorption of this drug. A limitation of this study is the lack of intravenous (IV) administration of this drug, which precluded determining intravenous-dependent parameters including, volume of distribution and clearance, as well as confirming that the terminal component of the plasma drug concentration vs. time curve reflected elimination (rather than absorption as occurs with a “flip-flop” model). There may be benefit to increase the frequency of dosing to provide a further steady state of mebendazole concentration in plasma and CSF, and would require further investigation to better understand the effect of food on absorption of this particular benzimidazole.

Another limitation to this study could be related to CSF collection and analysis. For this study, CSF samples were not analyzed for protein or cell counts (i.e., red blood cells or white blood cells) as the focus was on drug concentration. As such, some gross blood contamination was observed during the CSF collection period. To attempt to limit the effects of this, the sample was allowed to flow until grossly clear however it is unclear if this could impact achieved CSF concentrations nor if this could impact future samples. This occurred in a total of 5 dogs (*n* = 1 from the 100 mg/kg group at the 1-h time point and *n* = 4 from the 200 mg/kg group). For the dogs in the 200 mg/kg group, blood contamination occurred at the 2-h and 3-h time point in one dog, at the 3-h and 12-h point in one dog, at 12 h in one dog, and at sample points 3-, 6-, 12-, and 24- h in one dog. Due to the variability observed in the CSF concentrations in relation to the relative bioavailability of these samples, it does not appear obvious that blood contamination significantly contributed to these values.

From this study, the question remains if an *in vitro* IC_50_ will correlate with *in vivo* activity and if brain tissue concentrations will mirror the CSF concentrations as well to ultimately target the tumor cells. Although these two doses were able to achieve detectable CSF concentrations, optimal target concentrations in the CSF for treatment of canine gliomas remains to be determined and this does not support sustained CSF concentrations nor the ability to penetrate brain tissue rather than CSF. Determining whether or not we could achieve therapeutic concentrations of mebendazole within the plasma and CSF in healthy canines was the first step towards further investigating the use of this drug as a novel treatment for gliomas. Ideally, a follow up study to determine if consistent dosing of 200 mg/kg could consistently achieve CSF concentrations considered therapeutic in dogs with gliomas. However, given the limitation of patient access and ethical concerns regarding a pharmacokinetic study in client-owned dogs with a likely fatal CNS neoplasm, the data here may be sufficient to support a phase II efficacy study in afflicted dogs. Ultimately, the information from this study would be utilized to determine the efficacy of mebendazole as an adjunctive therapy for canine gliomas to prolong survival time in canines with this fatal disease.

## Data availability statement

The raw data supporting the conclusions of this article will be made available by the authors, without undue reservation.

## Ethics statement

The animal study was approved by Auburn University Institutional Animal Care and Use Committee. The study was conducted in accordance with the local legislation and institutional requirements.

## Author contributions

AY, KD, AT, and DB conceived and planned the experiments. AY and KD ran the experiments and began initial manuscript preparation. CC-E and DB analyzed the plasma and CSF samples. AY and DB analyzed the data. All authors contributed to the article and approved the submitted version.

## Funding

This study was funded by the Auburn University Intramural Animal Health and Disease Research (AH&DR) seed grant.

## Conflict of interest

The authors declare that the research was conducted in the absence of any commercial or financial relationships that could be construed as a potential conflict of interest.

The handling editor RB declared a past co-authorship with the author AY.

## Publisher’s note

All claims expressed in this article are solely those of the authors and do not necessarily represent those of their affiliated organizations, or those of the publisher, the editors and the reviewers. Any product that may be evaluated in this article, or claim that may be made by its manufacturer, is not guaranteed or endorsed by the publisher.
